# Vancomycin-resistant enterococci: eradication using vancomycin?

**DOI:** 10.1186/cc13546

**Published:** 2014-03-17

**Authors:** J Van Prehn, AM Stemerding, HK Van Saene, PE Spronk

**Affiliations:** 1 VU University Medical Center, Amsterdam, the Netherlands; 2 Gelre Hospitals, Apeldoorn, the Netherlands; 3 University of Liverpool, UK

## Introduction

Patient-to-patient transmission enables vancomycin- resistant enterococci (VRE) outbreaks. Outbreak management is expensive and time consuming, and therefore the possibility of VRE eradication is desirable. Since vancomycin is scarcely absorbed in the gastrointestinal tract, treatment with vancomycin *per os *may result in very high gastrointestinal concentrations (many times the minimum inhibiting concentration (MIC)). The purpose of this study is to measure *in vivo*gastrointestinal concentrations of vancomycin in patients that are treated with a standard dose orally, and to investigate *in vitro *whether vancomycin is able to kill VRE at concentrations up to 2,000 times the MIC.

## Methods

The faecal vancomycin concentration was measured in eight patients who suffered a *Clostridium difficile *infection and were treated with 4 × 500 mg vancomycin orally per day. *In vitro, *a (1:2) dilution series of vancomycin (range 6,250 to 0.4 μg/ml) was created and 1 ml vancomycin solution was then added to 1 ml standardized inoculum. One vancomycin-susceptible enterococcus isolate (VSE, MIC = 3 μg/ ml) and two VRE isolates (MIC = 16 μg/ml) were studied. After 1, 7 and 14 days incubation at 35°C, growth was defined as macroscopic visible turbidity. To test for surviving bacteria, all inocula were cultured to sheep blood agar plates, which were read after 24-hour incubation at 35°C. E-tests to measure MIC were performed on relevant samples. The *in vitro *experiment was performed twice.

## Results

The faecal vancomycin concentration in patients treated orally with vancomycin was 8,000 μg/ml on average. VSE growth at day 14 was detected at up to 1.5 μg/ml vancomycin, whereas VRE growth was detected at up to 98 μg/ml. The MIC of these VRE species growing at 98 μg/ml vancomycin was increased (≥256 μg/ml). For both VSE and VRE, surviving bacteria were detected at very high concentrations of vancomycin (>98 μg/ml): the MIC of these survivors was not increased.

## Conclusion

Oral treatment with vancomycin results in extremely high faecal concentrations. At these high concentrations, VRE bacteria are killed *in vitro; *however, a minority of the VRE is able to survive. Vancomycin thus seems unsuitable for eradication. However, high concentrations of vancomycin dramatically reduce the bacterial VRE load. Therefore oral treatment with vancomycin may help to terminate VRE outbreaks: a dramatic reduction in bacterial load of the colonised patient will minimise the risk of patient-to-patient transmission.

